# Perspective: Fat Reduction Campaigns and Their Impact on Young Children—The Root Cause of the Obesity Epidemic?^☆^

**DOI:** 10.1016/j.advnut.2025.100576

**Published:** 2025-12-19

**Authors:** Marie-Françoise Rolland-Cachera

**Affiliations:** Sorbonne Paris Nord University and Paris City University, National Institute of Health and Medical Research (INSERM), National Research Institute for Agriculture, Food, and the Environment (INRAE), National Conservatory of Arts and Crafts (CNAM), Center for Research in Epidemiology and StatisticS (CRESS), Nutritional Epidemiology Research Team (EREN), F-93017, Bobigny, France

**Keywords:** obesity, undernutrition, children, early-life programming, first 1000 d, fat intake, hypothalamic axis, leptin resistance, energy metabolism, thrifty metabolism

## Abstract

After decades of rising obesity rates, key questions about the causes of this epidemic remain unanswered. One major puzzle is the discrepancy between increasing obesity prevalence and decreasing energy and fat intake. Campaigns to restrict fat intake to prevent obesity have led to a decrease in fat consumption; however, little attention has been paid to the specific effect of this advice in early life. This review aims to evaluate the impact of such campaigns on the dietary habits and future health of young children, and to investigate the potential contribution of early nutritional changes to the obesity epidemic. Available data reveal that children’s fat intake has fallen drastically in recent decades, often reaching levels well below official recommendations. Reducing fat intake is not appropriate in early life, when fat is essential for brain development and meeting high energy needs. Early energy restriction can alter the hypothalamic axis, leading to a reduction in leptin level and irreversibly imprinting a “thrifty metabolism.” These mechanisms may decrease basal energy expenditure, develop leptin resistance, and promote fat storage. The unexpectedly high number of structural, functional, and metabolic similarities between undernourished individuals and subjects with obesity suggests a role of early energy restriction in programming obesity. A person with obesity can then be considered an undernourished individual covered with high-fat stores that cannot easily be used. By affecting the early period of life, advice to reduce fat intake to combat obesity may instead have contributed to its increase. This shift in thinking, from overnutrition to undernutrition, to explain the origin of obesity, highlights that fat intake should not be restricted in young children. The novel hypothesis that fat reduction campaigns, by affecting early life, could be a cause of the obesity epidemic should open new avenues for research and prevention.

**Statement of Significance**

Campaigns to restrict fat intake to prevent obesity have led to a decrease in fat consumption, whereas obesity prevalence was increasing. This decline in fat intake has particularly affected young children, programming a thrifty metabolism, which could be responsible for the obesity epidemic.

## Introduction

Since the origin of Homo sapiens, one of mankind’s main concerns has been the search for food, and populations had to cope with famines. However, after eras of scarcity, a global obesity epidemic has emerged in recent decades [1]. Despite significant progress in research, key questions remain about the root causes of this epidemic [2]. Surprisingly, although most research focuses on the determinants of excessive intake, obesity rates have risen significantly without evidence of increasing energy and fat consumption [3], but rather despite a decline over the last 4 decades [2,[4], [5], [6]]. This paradox is often interpreted as a consequence of reduced physical activity [3,4], but recent data rule out this interpretation [7].

The trend toward reducing fat intake, which has particularly affected young children, is in line with public health recommendations targeting obesity [6,8]. However, this strategy is poorly supported as many studies fail to show an association between fat intake and overweight in adults [[8], [9], [10]] and children [[11], [12], [13], [14], [15], [16], [17]]. Various factors, such as diet, physical activity or the social environment [2,[18], [19], [20], [21], [22]] are associated with the risk of obesity or metabolic diseases, but the growing number of observations linking undernutrition with obesity has prompted a major shift in thinking from overnutrition to undernutrition to explain body fat development [[23], [24], [25], [26]]. The imprinting of a “thrifty metabolism” during early life was proposed a long time ago to explain the link between early restrictions and the metabolic syndrome [27]. The mechanisms that may explain the association between early fat restrictions and obesity involve energy and hormonal responses. However, despite these widely recognized mechanisms, little attention has been paid to the consequences of recommendations to reduce fat intake on young children’s health.

The assessment of changes in nutritional intakes and body characteristics in recent decades has led to novel hypotheses about the consequences of fat reduction campaigns on the global obesity epidemic.

## Current Status of Knowledge

### Characteristics of nutrient intake in early life

The first 2 y of life are characterized by high energy requirements and are critical for the child’s neural development. The high-fat content of human milk is well suited to meeting these challenges [28,29]. The FAO provides science-based guidance on food and nutrition to national governments and the international community [28]. According to FAO, dietary fat should contribute 40% to 60% of energy intake during the first 6 mo of life, gradually decrease to 35% from 6 to 24 mo of age, and remain at this level into adulthood. Breast milk composition is also considered as a reference. However, in the last decades, these recommendations have often been ignored, and infants’ fat intake is usually very low in many populations [30]. Low-fat diets have been recorded in various European countries showing that, by the age of 1 y, children consume as little as ∼29% of their daily energy intake as fat [31]. Data from Spain, Sweden, France, Italy, Denmark, Finland, The Netherland, Belgium, and from the United Kingdom, and the United States [[32], [33], [34], [35], [36], [37], [38], [39], [40], [41]] are presented by age in Table 1.

**TABLE 1:** Nutritional intakes in early life during the last decades when the obesity epidemic was on the rise, compared with breast milk composition [28] and security intake [42]

Nutritional references and countries	Age	Intakes			
		Proteins	Proteins	Fat	CHO^1^
	Mo	g/kg/d	% Energy	% Energy	% Energy
Breast milk [28]	—	—	5	50	45
Security intake [42]	12	1.17	—	—	—
Country					
United Kingdom [40]	8	—	13.4	35.4	50.7
Spain [32]	9	4.4	15.7	26.4	57.9
Sweden [33]	9	—	15.0	28.0	55.0
France [34]	10	4.3	15.6	27.4	57.0
Italy [35]	12	5.1	19.5	30.5	50.0
Denmark [36]	12	3.3	15.0	28.0	57.0
Finland [37] 2	13	—	17.0	28.0	55.0
The Netherlands [38]	16	3.7	16.8	28.5	54.7
United States [41]	12–23	4.1	15.5	32.9	53.0
Belgium [39]	12–24	3.8	15.8	29.2	55.0
Mean intakes	—	4 g/kg/d	16%	29%	55%

1 Carbohydrates.

2 Data from control group.

The prevalence of children having too low intakes varies, depending on the recommendations. According to United States Dietary Guidelines, 12–23-mo old toddlers from the NHANES consumed an average of 32.9% of energy as fat, which is within the Acceptable Macronutrient Distribution Range (30%–40% Energy), and 28% of them had fat intakes that fell below this recommendation [41]. A recent review examined studies conducted from 2003 to 2017 [43] and evaluated intakes against FAO/WHO [28] and the European Food Safety Authority guidelines [44]. This review, including 65 studies from 33 countries found that, in children aged 1–3 y, fat intake was too low in 88% of studies. In the French Etude des Determinants pre et post-natals de la santé de l'Enfant (EDEN) study on nonbreast-fed children, mean percentage of fat intake was 31.4% at 8 mo and 29.7% at 1 y [45]. According to the same guidelines, 95% of 8-mo-old infants consumed <40% of their energy as fat. These studies suggest that a large proportion of children consume less than recommended amounts of fat despite the almost universal agreement that dietary fat should not be reduced during the first years of life [30].

In addition to low-fat intake, protein consumption by the age of 1 y reached as much as 16% of energy (Table 1), corresponding to ∼4 g/kg/d [31], which is ∼3 to 4 times the protein requirements [42]. The low-fat (29%) high-protein (16%) energy content of the diet typically consumed by children by the age of 1 y stands in striking contrast to the high-fat (50%)-low protein (5%) content of human milk whose composition may contribute to its widely recognized beneficial effects [[46], [47], [48], [49]].

### Trends in nutritional intakes

In recent decades, although the obesity epidemic was on the rise, energy intake decreased [5,6] but the nutrient content of the diet did not vary uniformly over time [34,46,[50], [51], [52], [53], [54], [55]] (Table 2).

**TABLE 2:** Trends in child nutritional intakes over the past decades

Country	Date	Age	Energy	Protein	Fat	CHO
		Y	kcal	% Energy	% Energy	% Energy
United States [46]	2002–2008	0.5–2	↓	↑1	↓2	↓
England [50]	1967–1993	1.5–2.5	↓	↑	↓	↔
France [34]	1973–1986	2	–	↑	↓	↔
Germany [51]	1985–2000	2–3	↔	–	↓	↑
United States [52]	1974–1994	2–5	↔	–	↓	↔
England, Scotland, Wales [53]	1950–1993	4	↓	↔	↓	↑
United States [54]	1973–1988	10	↔	↑	↓	↑
France [55]	1978–1995	10	↓	↑	↓	↔

Total energy and % energy from fat decreased, whereas protein generally increased. No consistent trend was observed for carbohydrates (CHO).

^1^Increasing number of United States toddlers consuming amounts above acceptable macronutrient distribution ranges.

^2^Increasing number of United States toddlers consuming amounts lower than acceptable macronutrient distribution ranges.

↑ Increased consumption.

^↔^ No change.

↓ Decreased consumption.

#### Changes in fat intake

Generally, both fat and energy intake decreased over the last decades, particularly in young children (Table 2). Among 1.5- to 2.5-y-old children in the United Kingdom [50], the proportion of energy from fat dropped from 38.3% to 36.4% between 1967 and 1993. Among 2-y-old French children, it decreased from 36.5% to 32.4% between 1973 and 1986 [34]. Similarly, in 2- to 5-y-old United States children, it decreased from 36.2% to 32.8% between 1971 and 1988 [52]. Reported decreases in fat and energy intakes are sometimes interpreted as a consequence of underreporting, but this bias has not been thoroughly investigated in children [52].

A decrease in dietary fat may have been achieved by limiting added fat or by using low-fat dairy products, such as semi-skimmed milk, which contains 30% of its energy as fat, compared with 50% in whole milk. Children’s low-fat consumption is consistent with the general shift from using full-fat to low-fat dairy products [6,56], a practice that particularly affects young children who consume large amounts of dairy products [17,30,34]. This trend follows campaigns to reduce fat intake which stems from the controversial belief that fat intake is associated with obesity and metabolic diseases [[8], [9], [10],^57^]. Young children are likely to experience a major reduction in fat energy intake because their diet is wholly dependent on adults [2] who are often concerned that the child may become obese or develop atherosclerosis. This belief comes from misconceptions regarding infant feeding [58,59]. Furthermore, during early childhood, it is easy to intervene in a diet consisting mainly of dairy products. The shift from whole milk to low-fat milk could be offset to some extent by infant formulas which have a more adequate fat and protein content, but are still not equivalent to human milk [60] and are used only for a short period of time [13]. The widespread use of infant formulas nowadays could have improved young children’s intake. After a significant reduction in fat intake since the 1970s, recent reports indicate that the decrease has attenuated since 2000 [61,62], whereas the prevalence of obesity has stabilized [63]. However, most recent studies show that fat intake remains below recommendations in a majority of infants [43,45].

The earlier introduction in the diet of cheaper, unmodified, usually fat-reduced cow’s milk, which is currently used by families of lower economic status [64], may explain the lower fat content of diets provided by mothers with shorter education [45]. The choice of milk for infants, like the quality of food in the general population, can be driven by price and may contribute to the social disparities in obesity rates [20].

The consequences of low-fat intake were initially studied in the context of deficiencies in developing countries [65], but concerns about growth failure also arose in relation to fat restriction [58,66]. Low-fat diets decrease energy intake [28] but they do not necessarily affect growth [37,46]. An intervention study conducted in 7-mo-old infants [37] concludes that infants consuming 26% of energy as fat experienced no growth impairment and that 25% to 30% energy as fat is an acceptable range for preventing cardiovascular risk. This result was to be expected, as the decrease in lipids was offset by an increase in growth-stimulating proteins that accounted for 18% of energy, a very high level compared with 5% in human milk. Even if it does not compromise growth, low-fat intake can have other adverse consequences by altering the nervous system development, because lipids are a structural component of all tissues [28,29]. The rationale behind limiting infants’ fat intake has been questioned, given the high (mainly saturated) fat content of human milk [60,67], and because most studies do not support the notion that children’s fat intake increases the risk of developing adiposity and cardiovascular diseases [11,[15], [16], [17]]. In addition, cholesterol was reduced in the intervention group. However, breastfed infants adapt to the high cholesterol content of human milk through a decrease in cholesterol synthesis [66], which may imprint lower cholesterol production and levels in later life.

#### Changes in protein intake

Studies on secular trends report an increasing amount of protein in children’s diet, particularly in infants and toddlers, alongside a decrease in fat intake (Table 2). This may be due to the selection of low-fat dairy products such as semi-skimmed milk, which contains 30% of energy as protein, as opposed to 20% in whole milk [6,56], a very high level compared with 5% in human milk.

The role of protein intake was initially studied in the context of deficiencies [65]. The consequences of excessive protein intake have emerged more recently. Almost 3 decades ago, an association between high-protein intake in early life and increased fatness development, particularly at the abdominal site, was first reported in the 2-decade-long French Etude Longitudinale Alimentation Nutrition Croissance des Enfants (ELANCE) study [68]. This association now referred to as the “early protein hypothesis” has been subsequently supported by many observational studies [69,70]. The causal link between an early excess of protein and body adiposity was confirmed in a large, double-blind randomized trial [71]. The hypothesized mechanism [68] was that high-protein intake increases insulin-like growth factor-1 (IGF-1) levels, thereby stimulating growth and triggering adipocyte multiplication [72,73]. In the intervention study, IGF-1 increased in children fed a high-protein diet [74], whereas similar weight trajectories were reported between the breastfed and the low protein groups [71]. The quality of protein also matters. Animal proteins, specifically milk proteins, increase IGF-1 and stature [75]. This effect is particularly important in infants whose diet mainly consists of dairy products. The high-protein diet explains the rapid growth, early maturation, increased stature and lean body mass, as well as the early adiposity rebound, characteristics of children with obesity [72,[76], [77], [78], [79]].

#### Changes in carbohydrate intake

Unlike energy and lipid intakes (which decrease consistently) or protein intake (which increases consistently), trends in carbohydrate (CHO) intake are inconsistent [6] (Table 2). However, when it comes to CHOs, it is important to distinguish between complex CHOs and sugar. There is broad agreement about the association between excessive intake of added sugar and metabolic dysfunction [57,80,81]. Nevertheless, despite the expected increase, a general decrease in sugar consumption has been observed in recent decades, including in sugar-sweetened beverages [6,46,53,54,82]. This trend is mainly due to decreased consumption of table sugar, which is partially offset by increased consumption of sweet bakery products and ultra-processed foods [6,82]. Sugar consumption appears to have a specific effect during the first years of life. A study examining the impact of limited exposure to sugar during the rationing period of the 1950s in the United Kingdom found that sugar rationing in early life reduced later metabolic dysfunction, whereas later high-sugar intake increased metabolic diseases, suggesting the existence of a biological pathway induced by a high-glycemic diet [57].

#### Changes in nutrient partitioning in young children compared with adults

Although energy intake has decreased in recent decades, the nutritional composition of diets did not vary uniformly and changes differed between adults and children. Logically, a proportional decrease in 1 nutrient leads to increases in other nutrients, creating uncertainty about the factors affecting health in adults [57] and children [17]. It has been suggested that the public health recommendations to reduce dietary fat have led to an increase in CHO consumption [57]. The replacement of dietary fat with CHO in adults contrasts with the situation in children. The large amounts of dairy products consumed by young children have led to the replacement of fat by protein, rather than CHO (Table 2). Replacing fat intake with other nutrients can drive hormonal responses, such as increased growth factors [68,75] or insulin [57]. According to the “CHO-insulin model,” the increased glycemic load shifts metabolism toward fat storage in adipose tissue, thereby contributing to increased obesity rates [57]. Therefore, recommendations to decrease fat intake should consider the risks associated with its replacement by other nutrients.

### Nutritional factors affecting energy balance and growth

#### Factors driving energy reduction

Both low-fat and high-protein intake affect the energy balance, albeit in different ways. A low-fat diet results in lower energy intake in children [28,66]. Limiting fat intake reduces the energy density of the diet, which is unsuitable for young children with small stomachs [28]. High-protein intake limits available energy input by: *1*) increasing “Dietary Induced Thermogenesis” due to the higher energy cost of digestion, absorption, and metabolism, compared with high-fat or high-CHO diets [83], *2*) decreasing energy intake according to the “protein leverage model,” since consuming protein-dense foods, such as low-fat dairy products, requires smaller quantities to meet the body’s protein requirements [84], and *3*) increasing energy needs due to accelerated growth [70,75]. Given the multiple effects of the nutrient composition of the diet on energy balance, it is important to emphasize that the energy deficit in early life is not only due to the reduction in fat and energy intake, but also to a number of nutrient-specific effects on metabolism and growth.

#### Microbiota and energy metabolism

There is growing evidence that the human colonic microbiota can contribute to host nutrition and health. Alterations in the gut microbiota in early life can increase the risk of obesity by influencing nutrient absorption and metabolism, as well as gut–brain communication [85]. Given the importance of fat intake for brain development [28,29] and the high-fat content of human milk, fat intake in early life may positively affect the brain–gut axis. This neurohumoral communication system between the gastrointestinal tract and the brain is an important signaling axis for maintaining the metabolic balance in humans [85]. Reduced-fat intake in early life could alter this process and may explain the lower quality of the microbiota in non-breast-fed infants [86].

#### Effects of nutritional intake on growth

The selection of high-protein, low-fat foods in industrialized countries contrasts with the low protein diet available in low-income countries, which slows children’s growth and saves energy [24,65]. Both “stunting” (short stature) in developing countries and “bolting” (tall stature) in industrialized countries can be seen as adaptive mechanisms for coping with protein-energy deficit or excess, respectively [77]. Faltering growth due to poor perinatal nutrition [65], or rapid growth as observed in childhood obesity [72,78,79] may offer short-term benefits but increase long-term metabolic risks [22,24,25,78,87,88].

**FIGURE 1:**
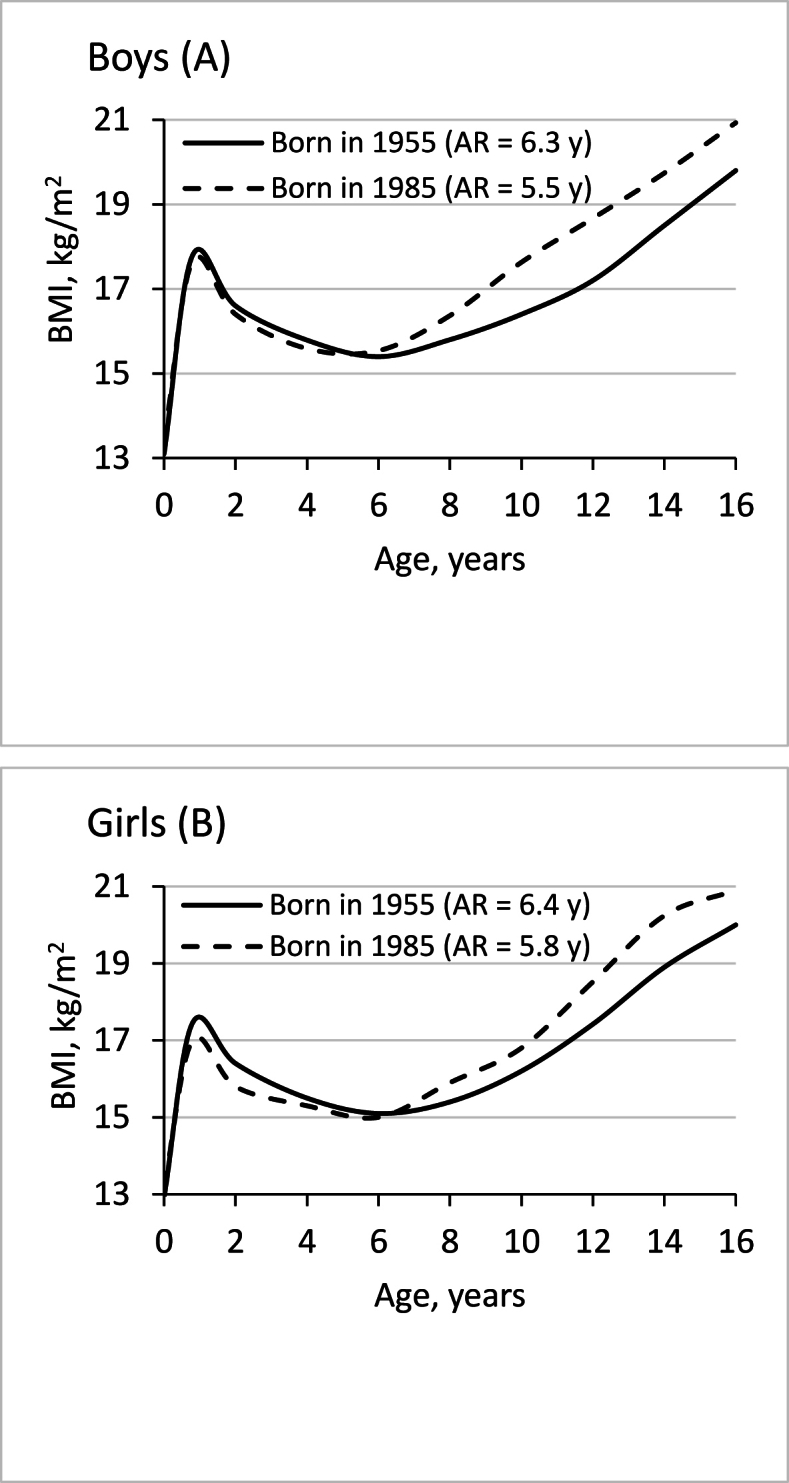
Trend in mean BMI trajectories and age at adiposity rebound (AR) of boys (A) and girls (B) from 2 cohorts of French children born 30 y apart [34]. In the more recent sample, BMI was lower during the first years of life, followed by an early AR and subsequently higher BMI values. This is the typical BMI trajectory observed in subjects with metabolic diseases such as diabetes [90,91]. This trend in BMI trajectories is likely to be a consequence of increased protein intake and decreased fat intake in early life.

The health effects of changes in nutritional intake can be evidenced by examining growth patterns recorded over different time periods. Compared with children of the Fels longitudinal study born between 1929 and 1953, the BMI trajectory of children born 40 y later (between 1973 and 1999) was characterized by significantly lower mean BMI values up to the age of 5 y, an earlier adiposity rebound, and more rapid BMI gain from the age of 8 y onward [89]. Similar changes in BMI patterns were observed between 2 cohorts of French children born 30 y apart [34] (Figure 1). The unexpected decreased BMI in early life is not the consequence of reduced weight, because both weight and height increased between the 2 periods. Rather, it is the result of a more rapid increase in height than weight [77], probably as a consequence of increasing protein intake over time. According to the early imprinting concept, the resulting low weight-for-height in early childhood may have programmed the development of obesity [27,87].

Both high-protein and low-fat dietary intakes in early life affect growth, but with different timing [92]. The effect of high-protein intake appears in the short term (rapid growth, early adiposity rebound) [68], and is likely due to an increase in growth factors [75]. In contrast, the consequences of early fat restriction increasing body fat only appear in the long term, likely by developing leptin resistance [13].

The early energy deficit due to low-fat, high-protein diet, followed by the exposure to an obesogenic environment [2], is the likely cause of the change in growth patterns and increased obesity over time. The BMI trajectory characterized by a low BMI in early life, followed by an early adiposity rebound and a subsequently high BMI, is the typical growth pattern of individuals at risk of obesity [77] and metabolic diseases such as diabetes [90,91]. The trend of lower BMI levels in children under 5 y in recent years could explain why the absolute rate of increase in overweight is lower among children than among adults in most countries [1,93].

### Similarities between undernutrition and obesity

Suboptimal nutrition in early life may be the link between the many abnormalities unexpectedly found in both subjects with obesity and undernourished individuals. Both conditions are associated with similar physiological dysfunctions [23,[94], [95], [96], [97], [98]], altered body composition [24,65,[99], [100], [101]], and food preferences [102]. Disorders include immune deficiency [103], impaired reproductive function [23,98], insulin resistance [94], euthyroid sickness state [23,65], elevated plasma free fatty acid levels and high hypothalamic concentration of neuropeptide Y [99], fat accumulation in the liver (mainly as triglycerides) [100], increased levels of circulating glucocorticoids [65], increased total body water and extracellular/intracellular water ratio [65], decreased sodium pump activity [101], impaired glucose tolerance [97], inflammation and android body fat distribution [24], increased appetite with a marked preference for fat [88,102], and decreased leptin activity (low level or resistance) [23,95,96].

Preferences for energy-dense foods may be driven by survival [102], thus raising the question of the contributions of hedonic (preference or “liking”) and homeostatic (physiological “wanting”) motivations for food consumption [104]. Wanting is more likely to be a response to high energy needs in both undernourished individuals and subjects with obesity. An android body fat distribution in obesity may result from the imprint of early undernutrition, which favors the reduction of peripheral fat while preserving abdominal fat, as it is essential for immunity and survival [24]. The many similarities between undernutrition and obesity support the notion that early energy restriction is the common factor for the development of obesity. A person with obesity can then be considered an undernourished individual covered with large fat stores that cannot be used readily. Therefore, I propose the concept of “undernourished individuals coated with fat” to describe persons who have become obese due to early energy deprivation.

### Early energy restrictions: metabolic consequences

Both low-fat and high-protein intake in early life contribute to an energy deficit, which has long-term health consequences [[24], [25], [26],^78^,^79^,^87^,^105^]. The association between energy restriction in early life and later metabolic disorders is consistent with observations reported in contexts related to energy deficiency, such as low birth weight [27,106], pregnancy during famines [107], seasonal food shortages [25], or poor nutrition in low-income countries [24,25,65,105,108]. Energy deprivation at any time during the “first 1000 d of life” (a critical window for future growth and development, from conception to 2 y of age) will imprint the risk of developing later obesity [87].

Early energy restriction, whether it results from adverse situations or from deliberate food choice in industrialized countries, is likely to be the common driver linking undernutrition and obesity [24]. Adaptive starvation responses to nutritional challenges [[24], [25], [26], [27],^78^,^88^,^105^,^109^] decrease energy expenditure, which promotes a prolonged positive energy balance. This is consistent with the decline in basal energy expenditure observed in recent decades [7].

**FIGURE 2:**
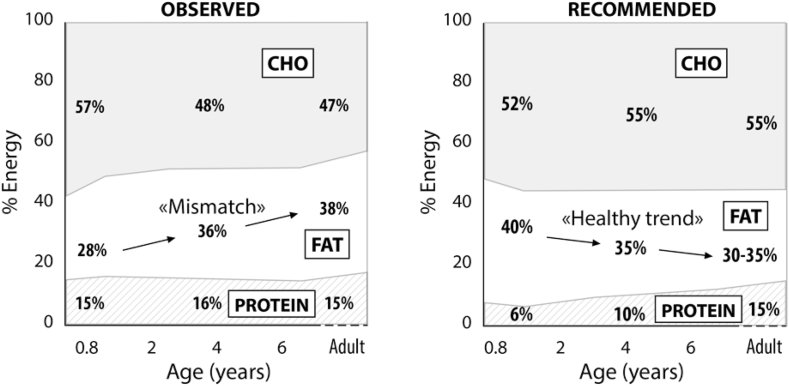
Observed (Left) and recommended (Right) carbohydrates (CHO), fat, and protein proportions throughout the life course: dietary intakes from the ELANCE longitudinal study [34] are compared with usual recommendations [28,44,110]. Reported fat intake is low during the “first 1000 d” sensitive period of life, and subsequently increases with age, whereas it should be high during early life and decrease subsequently, to meet nutritional requirements at the different stages of growth. Adapted from reference [92]. ELANCE, Etude Longitudinale Alimentation Nutrition Croissance des Enfants.

The contrast between increasing fat intake with age [92] and recommendation that fat intake should be high in early life and subsequently decrease [28] (Figure 2) illustrates the concept of a “mismatch” between an anticipated low-available energy supply and the actual “obesogenic” environment, which exposes the mature organism to the risk of adverse consequences [88]. Two studies illustrate the importance of nutritional intake trajectories. In the Avon Longitudinal Study of Parents and Children (ALSPAC) study, fat intake was close to the recommended level in early life and remained stable throughout childhood [40], whereas in the ELANCE study [13], fat intake was initially low and subsequently increased, in contrast with recommendations. The more favorable nutrient changes across ages in the ALSPAC study may explain why this study did not replicate the association between early fat restriction and higher body fatness in adulthood observed in the ELANCE study.

Another mechanism may contribute to the mismatch. The “protein leverage model” [84] proposes that the consumption of protein-diluted, highly processed foods increases the risk of overeating to meet the body’s protein requirements. By contrast, the protein leverage mechanism can decrease energy consumption in young children on high-protein diets and contribute to the mismatch between a low energy intake in early life followed by high energy intake.

The consequences of early undernutrition are consistent with the “adiposity-force theory,” which proposes that at any age the body responds to threats of future food shortages by building up a reserve of energy, independent of energy balance regulation, a mechanism that may favor obesity [111,112]. Similarly, the epigenetic selection of “thrifty” genes for survival in times of famine is likely to have contributed to the obesity epidemic [25].

### Energy restrictions: hormonal consequences

#### Leptin during perinatal life

Nutrition affects most hormones. Leptin, for example, is influenced by energy intake and plays an important role at different stages of life. In both humans and animals, energy restriction is associated with decreased leptin levels [23,24,95,106,113,114]. Friedman [23] proposes that the brain interprets a low leptin level as a signal of dangerous undernutrition and activates an adaptive “starvation” response. The positive response to leptin treatment in leptin-deficient patients confirms the physiological role of leptin [23]. Leptin-depleting undernutrition during the perinatal life can lead to metabolic disorders throughout life by affecting the hypothalamic pathways that regulate energy balance. A plethora of structural changes occurs, including alterations in neurogenesis, axon growth, and leptin receptor expression [23,98,113]. Consistent with the effects of leptin reduction in humans, blocking leptin action during the critical period of early life in rodents has long-lasting consequences by altering the ability to respond to leptin in adulthood, thus predisposing the animals to obesity [113]. In contrast to the adverse effects of reduced leptin in early life, the leptin content of human milk contributes to the health benefits of breastfeeding [115].

The long-term consequences of early fat restriction were demonstrated in the French 2-decade-long ELANCE study [13]. Low-fat intake at 2 y of age was associated with high abdominal fat and high leptin level at the age of 20 y, suggesting that early fat restriction can lead to leptin resistance. This association between an early energy deficit and later high leptin levels is consistent with previous findings of an association between low weight at birth [116] or at the age of 2 y [117], and high leptin level in adulthood. The ELANCE study also found that low-fat intake in early life was significantly associated with a decreased number of leptin receptors by the age of 20 y (Rolland-Cachera, unpublished data), a mechanism that may lead to leptin resistance. The low responsiveness to leptin administration in patients with obesity with elevated plasma leptin levels may be explained by reduced leptin receptors [23].

Leptin resistance is often viewed as a result of increased adiposity. This observation mainly derives from studies conducted in overweight adult populations [116,118]. In young children, fat restriction decreases leptin level, which may program leptin resistance and body fat development. Restoring leptin receptor function is then an interesting research avenue for addressing obesity.

Leptin resistance suggests that obesity is more likely to result from inhibited lipolysis and the difficulty of using fat stores, than from excessive lipogenesis caused by overeating. Reduced lipolysis explains why fat accumulation can occur without excessive energy intake, as evidenced by comparisons of lean compared with obese animals under controlled conditions, as well as in humans [23], including children [119].

#### Leptin and the obesity epidemic

On the basis of the links between fat restriction and obesity, I suggest that the obesity epidemic has emerged in a few stages. Campaigns to reduce fat intake have: *1*) reduced energy intake particularly in young children during the sensitive period of early life, *2*) altered hypothalamic functions and reduced leptin levels, and *3*) irreversibly imprinted a “thrifty metabolism,” thus programming leptin resistance and fat storage.

This simple process may account for the diverging trends between declining energy and fat intake compared with rising obesity prevalence. However, the notion that nutritionally imbalanced diets in early life affect leptin metabolism does not rule out disturbances in other hormonal systems [57,120].

### Energy metabolism and ethical considerations

Although weight status is the result of a balance between energy ingested and energy expended, obesity is often viewed as the result of excess energy intake. However, many observations suggest that early energy restrictions may result in undernourished body characteristics with impaired metabolic functions and limited access to body fat stores. Obesity should then be considered a medical condition that prevents the utilization of body fat, rather than the result of gluttony and lack of willpower [121].

In conclusion, obesity rates have risen sharply in recent decades despite campaigns to reduce energy and fat intake. The consequences of this strategy on the nutritional intakes and future health of children have received little attention. The striking reduction in fat intake during the “sensitive early period of life” may have altered children’s metabolic and hormonal status, decreasing their basal energy expenditure and thus promoting obesity. This mechanism may resolve the puzzling paradox of the increasing obesity prevalence despite no increase in energy intake. The shift in thinking from overnutrition to undernutrition to explain the development of obesity highlights the fact that fat intake should not be restricted in young children.

The hypothesis that fat reduction campaigns could be at the origin of the obesity epidemic by impacting early life should open up new avenues for research and prevention.

## Author contributions

The sole author was responsible for all aspects of this manuscript.

## Declaration of generative AI and AI-assisted technologies in the writing process

The author did not use generative AI and AI-assisted Technologies in the writing process.

## Funding

This research received no external funding.

## Conflict of interest

The authors report no conflicts of interest.
